# Roles of Macrophages in Advanced Liver Fibrosis, Identified Using a Newly Established Mouse Model of Diet-Induced Non-Alcoholic Steatohepatitis

**DOI:** 10.3390/ijms232113251

**Published:** 2022-10-31

**Authors:** Yuki Tada, Kaichi Kasai, Nana Makiuchi, Naoya Igarashi, Koudai Kani, Shun Takano, Hiroe Honda, Tsutomu Yanagibashi, Yasuharu Watanabe, Fumitake Usui-Kawanishi, Yukihiro Furusawa, Mayuko Ichimura-Shimizu, Yoshiaki Tabuchi, Kiyoshi Takatsu, Koichi Tsuneyama, Yoshinori Nagai

**Affiliations:** 1 Department of Pharmaceutical Engineering, Faculty of Engineering, Toyama Prefectural University, 5180 Kurokawa, Toyama 939-0398, Japan; 2 Toyama Prefectural Institute for Pharmaceutical Research, 17-1 Nakataikouyama, Toyama 939-0363, Japan; 3 Department of Pathology and Laboratory Medicine, Tokushima University Graduate School of Biomedical Sciences, 3-8-15 Kuramoto-cho, Tokushima 770-8503, Japan; 4 Division of Molecular Genetics Research, Life Science Research Center, University of Toyama, 2630 Sugitani, Toyama 930-0194, Japan

**Keywords:** Kupffer cell, fibrosis, inflammation, macrophage, non-alcoholic fatty liver disease, non-alcoholic steatohepatitis

## Abstract

Macrophages play critical roles in the pathogenesis of non-alcoholic steatohepatitis (NASH). However, it is unclear which macrophage subsets are critically involved in the development of inflammation and fibrosis in NASH. In TSNO mice fed a high-fat/cholesterol/cholate-based diet, which exhibit advanced liver fibrosis that mimics human NASH, we found that Kupffer cells (KCs) were less abundant and recruited macrophages were more abundant, forming hepatic crown-like structures (hCLS) in the liver. The recruited macrophages comprised two subsets: CD11c^+^/Ly6C^−^ and CD11c^−^/Ly6C^+^ cells. CD11c^+^ cells were present in a mesh-like pattern around the lipid droplets, constituting the hCLS. In addition, CD11c^+^ cells colocalized with collagen fibers, suggesting that this subset of recruited macrophages might promote advanced liver fibrosis. In contrast, Ly6C^+^ cells were present in doughnut-like inflammatory lesions, with a lipid droplet in the center. Finally, RNA sequence analysis indicates that CD11c^+^/Ly6C^−^ cells promote liver fibrosis and hepatic stellate cell (HSC) activation, whereas CD11c^−^/Ly6C^+^ cells are a macrophage subset that play an anti-inflammatory role and promote tissue repair in NASH. Taken together, our data revealed changes in liver macrophage subsets during the development of NASH and shed light on the roles of the recruited macrophages in the pathogenesis of advanced fibrosis in NASH.

## 1. Introduction

Non-alcoholic fatty liver disease (NAFLD) is primarily a manifestation of metabolic syndrome in the liver. NAFLD is a disease that encompasses a wide range of liver pathologies, from non-alcoholic fatty liver (NAFL) to non-alcoholic steatohepatitis (NASH), cirrhosis, and hepatocarcinoma [1]. The etiology of NAFLD is thought to involve both genetic predisposition and epigenetic regulation by environmental factors such as lifestyle [1]. The pathogenesis of NAFLD has been explained using the so-called “two-hit theory,” which divides the pathogenesis into a first step, which involves fat deposition in hepatocytes, and a second step, which involves the development of inflammation and fibrosis [2]. However, the multiple parallel-hit hypothesis, which states that multiple factors have a simultaneous role in the pathogenesis, has been more widely accepted [2]. NASH is histologically characterized by steatosis, lobular inflammation, hepatocellular ballooning, and fibrosis of the liver. In humans, fibrosis in NASH typically first appears in zone 3 as a “chicken-wire” pattern, which spreads to the portal area, ultimately leading to portal–portal and portal–central bridging fibrosis [3].

The macrophage is the major phagocytic cell type in the innate immune system and these cells are involved in the inflammatory processes that characterize lifestyle-related diseases, including NASH [4]. A number of macrophage subsets exist and are present in various tissues, where they play important roles. For example, CD11c^+^ M1 macrophages in adipose tissue are pro-inflammatory, whereas CD206^+^ M2 macrophages are anti-inflammatory [5]. In particular, unique histological structures, termed crown-like structures (CLSs), are formed when CD11c^+^ macrophages aggregate around dead adipocytes to scavenge the lipid droplets [6]. In humans, the abundance of CD11c^+^ macrophages in adipose tissue correlates with markers of insulin resistance [7]. Importantly, the livers of patients with NASH exhibit similar structures to adipose CLSs, termed hepatic CLSs (hCLSs), in which CD11c^+^ macrophages surround dead or dying hepatocytes containing large droplets [8]. There is evidence that hCLS formation is involved in the development of hepatocyte death-induced liver fibrosis [8,9]. Importantly, hCLSs characterize human NASH and are associated with the transition from simple steatosis to NASH, whereas they are rarely present in patients with chronic viral hepatitis [8,10,11,12]. Overall, these previous findings are consistent with the notion that CD11c^+^ macrophage infiltration into the liver contributes to hCLS formation and fibrosis in NASH. However, the functions of CD11c^+^ macrophages and their roles in the development of NASH are not fully understood.

There are two major types of macrophages in the liver: tissue-resident macrophages (Kupffer cells, KCs), and myeloid-derived macrophages that are recruited from the bone marrow and infiltrate the liver as part of an inflammatory process. KCs can be distinguished from recruited macrophages using the expression of various cell surface markers: KCs are F4/80^Hi^ and CD11b^Int^, whereas recruited macrophages tend to be F4/80^Int^ and CD11b^Hi^ [13]. Chemokine receptors, such as C-C chemokine receptor type 2 (CCR2) and CX3C motif chemokine receptor 1 (CX3CR1), are expressed on recruited macrophages, whereas KCs do not express these receptors [14]. Recently, KC-specific markers have been identified, which include TIM4 [15]. The importance of macrophage recruitment in the pathogenesis of fibrosis during murine NASH has been widely recognized [16,17,18,19,20]. The recruited macrophages, which express CCR2/CX3CR1, preferentially accumulate in hCLSs and are required for their formation and interaction with other recruited macrophages [21]. The administration of a combination of a CCR2/ C-C chemokine receptor type 5 (CCR5) inhibitor and a fibroblast growth factor 21 analogue reduces the recruitment of these macrophages and liver fibrosis in a murine model of NASH [22]. In addition, tissue macrophages and KCs have been implicated in the development of liver fibrosis [23,24]. Thus, the contributions of resident and/or recruited macrophages to the development of liver fibrosis in NASH remain controversial.

Animal models are important means of analyzing the pathogenesis of NASH and testing potential therapeutic agents. The frequent administration of chemicals and diets deficient in specific nutrients have been used to create models of NASH. For example, the administration of carbon tetrachloride (CCl_4_) induces liver fibrosis, followed by secondary hepatocyte necrosis, similar to that which occurs in human viral hepatitis [25]. Methionine and choline-deficient diets are one of the most frequently used means of inducing NASH in animals, but the models created do not reflect human dietary habits and induce weight loss in mice [26]. Therefore, mice with specific gene deficiencies ± the administration of toxic agents, have also been used as models of NASH, but these are artificial situations and do not accurately reflect the pathogenesis of human NASH [27,28,29]. Thus, the currently used models do not reflect human dietary habits and are not characterized by histological changes similar to those of human NASH, such as bridging fibrosis.

Because there are few rodent models of NASH that progress to stage 3 or higher liver fibrosis, it is difficult to model the advanced fibrosis that occurs in human NASH using animals. The Tsumura-Suzuki non-obese (TSNO) mouse is a control strain for Tsumura-Suzuki obese diabetes (TSOD) mouse, which was created to be a polygenic model of obesity and type 2 diabetes by the selective breeding of ddY mice [30]. The TSNO mouse was also created from ddY mice, but does not exhibit either obesity or diabetes [31]. Ichimura-Shimizu et al. reported that a high-fat/cholesterol/cholate-based (iHFC) diet that is not deficient in specific nutrients, such as methionine and choline, induces advanced fibrosis in a ”chicken-wire” pattern that spreads to the portal area of TSNO mice [32]. Furthermore, iHFC-fed TSNO mice exhibit stage 3 bridging fibrosis. These histological features clearly suggest that iHFC diet-induced NASH in TSNO mice may represent a more faithful model of human NASH. However, TSOD mice do not develop NASH when consuming the iHFC diet, despite developing obesity and diabetes [32]. To date, the subsets of macrophages that characterize TSNO mice and their role in the development of advanced fibrosis have not been defined.

The purposes of the present study were to characterize the dynamics of resident and recruited hepatic macrophages and to better understand their roles in the development of advanced liver fibrosis, using a newly established mouse model of diet-induced NASH. Flow cytometry revealed that the iHFC diet induces the accumulation of CD45^+^ leukocytes, whereas CD45^−^ non-parenchymal cells are less abundant in the liver of mice fed the iHFC diet. Of these CD45^+^ leukocytes, F4/80^Int^/CD11b^Int-Hi^ macrophages show substantial infiltration into the liver in response to iHFC diet-feeding. These recruited macrophages were found to consist of two distinct macrophage subsets: CD11c^+^/Ly6C^−^ and CD11c^−^/Ly6C^+^ cells. Histological analyses demonstrated that CD11c^+^ cells accumulate in a mesh-like pattern around the lipid droplets, constituting hCLSs. In addition, CD11c^+^ cells were found to colocalize with collagen fibers in the livers of the iHFC-fed mice, suggesting that this subset of recruited macrophages might promote advanced liver fibrosis in this model. In contrast, Ly6C^+^ cells were found to accumulate in doughnut-like inflammatory lesions, with a lipid droplet at the center of each. Finally, RNA sequence analysis revealed that these two macrophage subsets have distinct gene expression profiles. Moreover, gene network analysis indicates that CD11c^+^/Ly6C^−^ cells promote liver fibrosis/hepatic stellate cell (HSC) activation, whereas CD11c^−^/Ly6C^+^ cells play a role in anti-inflammation/tissue repair, similar to M2 macrophages. Thus, we report the roles of two macrophage subsets in the development of liver inflammation and advanced fibrosis in a mouse model of NASH.

## 2. Results

### 2.1. iHFC Diet-Feeding Induces Inflammation, Steatosis, and Hepatocyte Ballooning in the Livers of TSNO Mice

**Figure 1:**
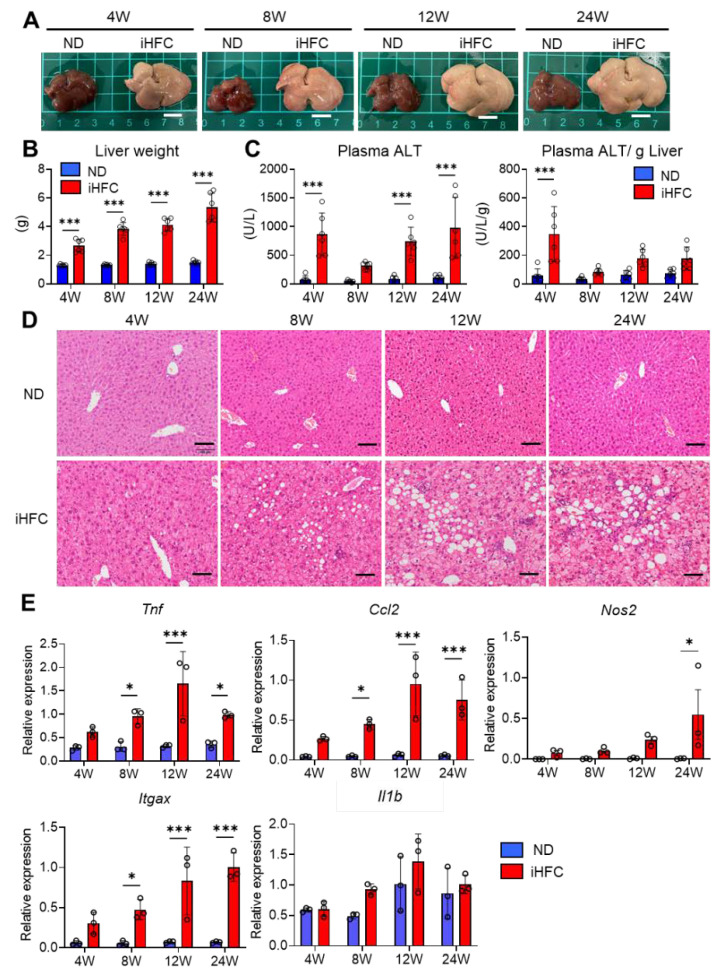
An iHFC (high-fat/cholesterol/cholate-based) diet induces steatohepatitis in the liver of Tsumura-Suzuki non-obese (TSNO) mice. (**A**) Representative photos of the livers from TSNO mice fed with the normal diet (ND) or iHFC diet for the indicated time periods. Scale bars, 1 cm. (**B**) Liver weights of TSNO mice fed with the ND or iHFC diet for the indicated time periods (*n* = 6 per group). (**C**) Left, plasma alanine aminotransferase (ALT) levels were measured for TSNO mice fed with the ND or iHFC diet for the indicated time periods (*n* = 6 per group). Right, plasma ALT levels per liver weight (g) were also calculated (*n* = 6 per group). (**D**) Representative images of hematoxylin and eosin-stained sections of the livers from TSNO mice fed with the ND or iHFC diet for the indicated time periods. Scale bars, 100 μm. (**E**) RT-qPCR of TNF-α (*Tnf*), iNOS (*Nos2*), CCL2 (*Ccl2*), CD11c (*Itgax*), and IL-1β (Il1b) mRNA in the livers from TSNO mice fed with the ND or iHFC diet for the indicated time periods (*n* = 3 per group). Data are shown as means ± SD. * *p* < 0.05, *** *p* < 0.001. Statistical significance was evaluated by 2-way ANOVA followed by post hoc Sidak test.

To investigate the roles of resident and recruited macrophages in the development of advanced fibrosis during NASH, we first confirmed that the iHFC diet induces inflammation and steatosis in the liver, as reported by Ichimura-Shimizu et al. [32]. Indeed, iHFC-fed mice had larger and paler livers than normal diet (ND)-fed mice (Figure 1A), and their liver masses significantly increased with the duration of consumption of the iHFC diet (Figure 1B). No significant differences were found in the body masses of mice consuming a ND or the iHFC diet (Figure S1A). In terms of average daily food intake, there was a significant difference between the ND and iHFC diet only in 4 weeks of feeding (Figure S1A). There was a transient increase in the plasma alanine aminotransferase (ALT) activities of the mice after 4 weeks of iHFC diet-feeding, and this was again high after 12 weeks (Figure 1C). The increase in plasma aspartate transaminase (AST) activities with the iHFC diet was similar to that of plasma ALT (Figure S1B). In addition, the iHFC diet significantly decreased the ratio of AST to ALT in plasma (Figure S1B). Mice consuming the iHFC diet also had high plasma total cholesterol (T-CHO) concentrations throughout the feeding period (Figure S1C). In contrast, the plasma triglyceride (TG) concentrations of the mice consuming the iHFC were much lower than those of mice consuming the ND (Figure S1C). Histopathological analysis revealed that mild lobular inflammation developed 4 weeks after the start of iHFC diet-feeding, and that this worsened as the duration of feeding increased (Figure 1D and Figure S2). In addition, steatosis and hepatocyte ballooning were observed from 8 weeks, and the grade of each increased with the duration of feeding (Figure 1D and Figure S2). These histopathological changes were not apparent in the ND group at either time point (Figure 1D and Figure S2). Furthermore, the expressions of a pro-inflammatory gene (Tnf), a chemokine gene (Ccl2, (C-C motif) ligand 2), and M1 macrophage marker genes (Nos2 and Itgax) were high after 4 weeks of iHFC diet-feeding (Figure 1E). No significant difference in IL-1β (Il1b) expression between ND and iHFC groups (Figure 1E). These data demonstrate that the iHFC diet induces inflammatory and pathological changes characteristic of NASH in the livers of TSNO mice.

### 2.2. iHFC-Fed TSNO Mice Develop Advanced Hepatic Fibrosis

**Figure 2:**
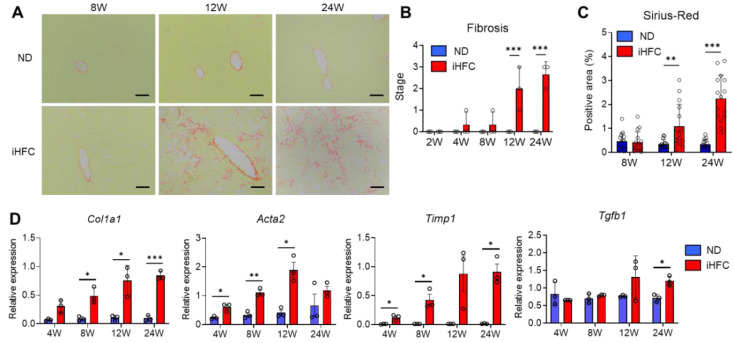
The iHFC diet induces advanced fibrosis in the liver of TSNO mice. (**A**) Representative images of Sirius red-stained sections of the livers from TSNO mice fed with the ND or iHFC diet for the indicated time periods. Scale bars, 100 μm. (**B**) Liver fibrosis (0 to 4) was assessed according to the criteria proposed by Kleiner et al., as described in the Materials and Methods (*n* = 3 per group). (**C**) Five locations were photographed per three sections per each group. Then, positive areas for Sirius red were measured at 15 locations using ImageJ software, and the mean and SD were calculated. (**D**) RT-qPCR of collagen type 1 (*Col1a1*), αSMA (*Acta2*), TIMP-1(*Timp1*), and TGF-β (*Tgfb1*) mRNA in the livers from TSNO mice fed with the ND or iHFC diet for the indicated time periods (*n* = 3 per group). Data are shown as means ± SD. * *p* < 0.05, ** *p* < 0.01, *** *p* < 0.001. Statistical significance was evaluated by 2-way ANOVA followed by post hoc Sidak test.

We also confirmed that the iHFC diet induces fibrosis in the livers of TSNO mice [32]. Perivenular and perisinusoidal fibrosis which was very similar to that observed in human NASH was present after 12 weeks of iHFC diet-feeding (Figure 2A,B). This fibrosis gradually expanded, with bridging fibrosis becoming apparent after 24 weeks of iHFC diet-feeding (Figure 2A,B). These fibrotic changes did not occur in the ND group (Figure 2A,B). The Sirius red-positive areas of the sections were also significantly higher than those of ND-fed mice from 12 weeks of iHFC diet-feeding (Figure 2C). Consistent with these data, the expression of collagen 1 and alpha-smooth muscle actin (αSMA) mRNA was higher in the livers of iHFC-fed mice (Figure 2D). The iHFC diet also markedly increased the mRNA expression of TIMP-1, which regulates extracellular matrix (ECM) degradation (Figure 2D). Indeed, high expression of these genes was present as early as 4 weeks after the start of iHFC diet-feeding (Figure 2D). In addition, the mRNA expression of TGF-β, which regulates ECM production, was high during the later stages of iHFC diet-feeding (Figure 2D). Thus, iHFC diet-fed TSNO mice develop advanced fibrosis in the liver that has an expanding pattern, similar to that of human NASH.

### 2.3. iHFC Diet-Feeding Causes the Accumulation of CD45^+^ Leukocytes in the Liver

**Figure 3:**
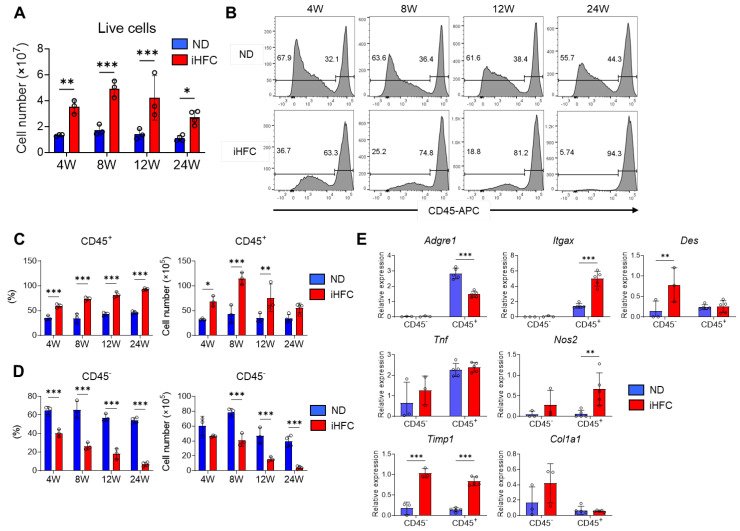
The iHFC diet induces the accumulation of CD45^+^ leukocytes in the liver of TSNO mice. (**A**) Cell number of live non-parenchymal cells of the livers from TSNO mice fed with the ND or iHFC diet for the indicated time periods (*n* = 3 or 4 per group). (**B**) Representative flow cytometry data of CD45 expression on live non-parenchymal cells of the livers from TSNO mice fed with the ND or iHFC diet for the indicated time periods. (**C**) Percentage (**Left**) and cell number (**Right**) of CD45+ live non-parenchymal cells were determined by flow cytometry analysis done in (**B**) (*n* = 3 or 4 per group). (**D**) Percentage (**Left**) and cell number (**Right**) of CD45- live non-parenchymal cells were determined by flow cytometry analysis done in (**B**) (*n* = 3 or 4 per group). (**E**) RT-qPCR of F4/80 (Adgre1), CD11c (Itgax), Desmin (Des), TNF-a (Tnf), iNOS (Nos2), collgen type 1 (Col1a1), and TIMP-1 (Timp1) mRNA in sorted CD45+ and CD45- cells from TSNO mice fed with the ND or iHFC diet for 8 weeks (*n* = 3 to 5 per group). Data are shown as means ± SD. * *p* < 0.05, ** *p* < 0.01, *** *p* < 0.001. Statistical significance was evaluated by 2-way ANOVA followed by post hoc Sidak test.

To investigate the roles of resident and recruited macrophages in the pathogenesis of iHFC diet-induced NASH, we isolated non-parenchymal cells from the liver and counted the numbers of CD45^+^ leukocytes at various time points of the feeding period. We found that TIM4-positive KCs were F4/80^Hi^/CD11b^Int^ (Figure S3). Because KCs are highly autofluorescent CD45^+^ cells (Figure S3), we used two different gating strategies, as shown in Figure S4, to quantify the CD45^+^ cells, depending on whether the KCs were being assessed or not. The number of live non-parenchymal cells significantly increased by iHFC diet-feeding (Figure 3A). Flow cytometric analysis demonstrated that the percentage of CD45^−^ cells was higher than that of CD45^+^ cells in the livers of the ND-fed mice, regardless of the duration of feeding (Figure 3B). In contrast, the iHFC diet-fed mice had a higher percentage of CD45^+^ cells, and this increased with the duration of feeding (Figure 3B,C). The number of CD45^+^ cells peaked after 8 weeks of iHFC diet-feeding and gradually decreased thereafter (Figure 3C). In contrast, the percentage and number of CD45^−^ cells markedly decreased with the duration of feeding (Figure 3B,D).

We then measured the expression of various genes in isolated CD45^+^ and CD45^−^ cells. The expression of F4/80 (Adgre1) and CD11c (Itgax) mRNA was predominantly in CD45^+^ cells (Figure 3E). The iHFC diet significantly increased the mRNA expression of CD11c in CD45^+^ cells and reduced that of F4/80 (Figure 3E). The iHFC diet also markedly increased the mRNA expression of desmin (Des), a marker of HSCs, in CD45^−^ cells (Figure 3E). The mRNA expression of TNF-α (Tnf) in CD45^+^ cells was higher than in CD45^−^ cells, and its expression was not significantly changed by iHFC diet-feeding (Figure 3E). iNOS (Nos2) and TIMP-1 (Timp1) were expressed at the mRNA level in both CD45^+^ and CD45^−^ cells, and the expression was increased by iHFC diet-feeding (Figure 3E). Collagen type 1 (Col1a1) mRNA expression in CD45^−^ cells was higher than in CD45^+^ cells and tended to increase with iHFC diet-feeding (Figure 3E). These data imply that CD45^+^ leukocytes, which include F4/80- and CD11c-expressing cells, accumulate with iHFC diet-feeding and play roles in inflammation and the degradation of ECM. In contrast, CD45^−^ cells, which include HSCs, are reduced in number by iHFC diet-feeding and are involved in fibrogenesis and ECM degradation.

### 2.4. An iHFC Diet Induces the Infiltration of the Liver with F4/80^Int^/CD11b^Int-Hi^ Macrophages, which Constitute hCLSs

**Figure 4:**
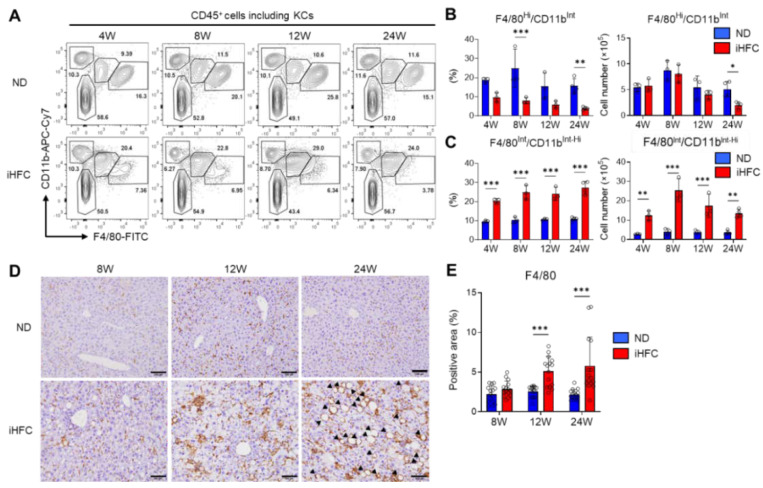
The iHFC diet accumulates F4/80^Int^/CD11b^Int-Hi^ recruited macrophages which constitute hCLS. (**A**) Representative flow cytometry data of F4/80 and CD11b expression on CD45^+^ non-parenchymal cells of the livers from TSNO mice on the ND or iHFC diet for the indicated time periods. (**B**) Percentage (**Left**) and cell number (**Right**) of F4/80^Hi^/CD11b^Int^ KCs were determined by flow cytometry analysis done in (**A**) (*n* = 3 or 4 per group). (**C**) Percentage (**Left**) and cell number (**Right**) of F4/80^Int^/CD11b^Int-Hi^ recruited macrophages were determined by flow cytometry analysis done in (**A**) (*n* = 3 or 4 per group). (**D**) Representative histological images of F4/80 immunostaining of the livers from TSNO mice on the ND or iHFC diet for the indicated time periods. Arrow heads depict hCLSs. Scale bars, 100 μm. (**E**) Five locations were photographed per three sections per each group. Then, positive areas for F4/80 were measured at 15 locations per each group using ImageJ software, and the mean and SD were calculated. Data are shown as means ± SD. * *p* < 0.05, ** *p* < 0.01, *** *p* < 0.001. Statistical significance was evaluated by two-way ANOVA followed by post hoc Sidak test.

We then focused on the subsets of CD45^+^ live non-parenchymal cells in the livers of iHFC-fed TSNO mice. At least three F4/80 and/or CD11b-expressing populations were present in ND-fed mouse livers: F4/80^−^/CD11b^Hi^ neutrophils, F4/80^Int^/CD11b^Int-Hi^ recruited macrophages, and F4/80^Hi^/CD11b^Int^ KCs (Figure 4A). There were no obvious differences in the percentages of F4/80^−^/CD11b^Hi^ neutrophils between the ND and iHFC groups (Figure 4A). Gr-1 staining also showed that neutrophils (CD11b^Hi^/Gr-1^Hi^) were present in the liver, and that there was no difference in their percentages between ND and iHFC diet-fed mice (Figure S5A,B). The percentage of F4/80^Hi^/CD11b^Int^ KCs was reduced by iHFC diet-feeding (Figure 4A,B), whereas the number of these cells significantly decreased only after 24 weeks of consumption of the iHFC diet, in comparison to the ND group (Figure 4B).

TIM4 is a specific marker of KCs [15]. In a mouse model of NASH, TIM4-negative monocyte-derived cells accumulate in the liver and take on the majority of features of KCs during the development of NASH [21]. TIM4 was highly expressed on F4/80^Hi^/CD11b^Int^ KCs from TSNO mice under normal conditions (Figure S6A), but consistent with the previous findings [21], there was a marked increase in the percentage of TIM4-negative KCs with the duration of feeding of the iHFC (Figure S6A,B). Indeed, by 24 weeks of iHFC-feeding, nearly 80% of the KCs were TIM4-negative (Figure S6A,B). In contrast, the number of TIM4-positive KCs was reduced by iHFC diet-feeding (Figure S6A,B). iHFC diet-feeding also markedly increased the number of F4/80^Int^/CD11b^Int-Hi^ recruited macrophages throughout the feeding period (Figure 4C), with a peak at the 8-week time point (Figure 4C).

The phosphorylation of p38, which promotes the progression of steatohepatitis by inducing pro-inflammatory cytokine secretion and M1 polarization, occurred much earlier in iHFC diet-fed mice, after 4 weeks (Figure S7) [33]. Immunohistochemical staining showed that the F4/80^+^ area of liver sections was gradually increased by iHFC-feeding (Figure 4D,E). Furthermore, hCLSs were apparent after 24 weeks (Figure 4D). Because the percentage of F4/80^Hi^/CD11b^Int^ KCs was markedly reduced by iHFC-feeding (Figure 4A,B), the F4/80^+^ area mostly reflected F4/80^Int^/CD11b^Int-Hi^ macrophage recruitment. These results imply that that iHFC diet induces hepatic infiltration with F4/80^Int^/CD11b^Int-Hi^ macrophages, which constitute hCLSs, suggesting that this macrophage subset might be involved in the development of hepatocyte death-induced liver fibrosis in this model of NASH.

### 2.5. F4/80^+^ Recruited Macrophages Include Two Macrophage Subsets, CD11c^+^/Ly6C^−^ and CD11c^−^/Ly6C^+^ Cells, in the Livers of iHFC-Fed Mice

**Figure 5:**
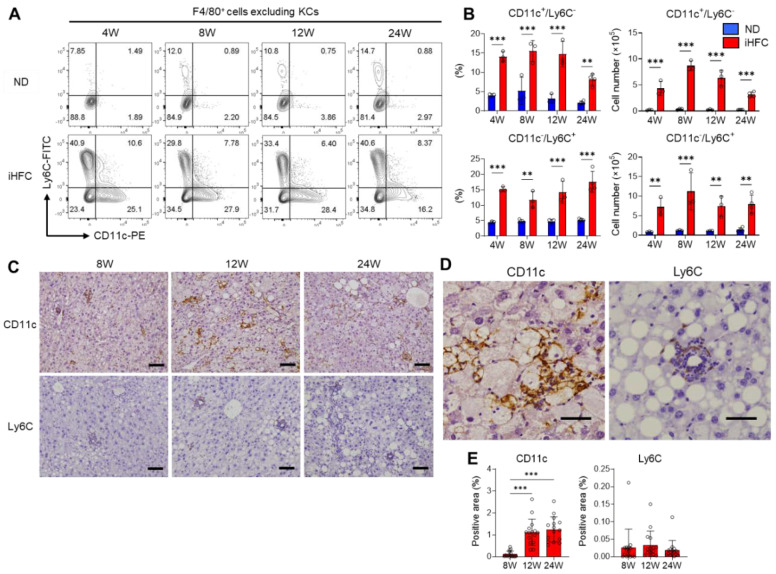
The iHFC diet accumulates two types of recruited macrophage subsets, CD11c^+^/Ly6C^−^ and CD11c^−^/Ly6C^+^ cells. (**A**) Representative flow cytometry data of CD11c and Ly6C expression on F4/80^+^ non-parenchymal cells excluding KCs of the livers from TSNO mice on the ND or iHFC diet for the indicated time periods. (**B**) Percentage and cell number of CD11c^+^/Ly6C^−^ or CD11c^−^/Ly6C^+^ were determined by flow cytometry analysis done in (**A**) (*n* = 3 or 4 per group). (**C**) Representative histological images of CD11c and Ly6C immunostaining of the livers from TSNO mice on the ND or iHFC diet for the indicated time periods. Scale bars, 100 μm. (**D**) Representative magnified images of CD11c and Ly6C immunostaining of the livers from TSNO mice on the iHFC diet for 24 weeks. Scale bars, 50 μm. (**E**) Positive areas for CD11c or Ly6C in the livers from TSNO mice on the iHFC diet for the indicated time periods were measured using ImageJ software. Data are shown as means ± SD. ** *p* < 0.01, *** *p* < 0.001. Statistical significance was evaluated by two-way ANOVA followed by post hoc Sidak test.

To identify the cell populations that comprise the F4/80^Int^/CD11b^Int-Hi^ recruited macrophages, we gated F4/80^+^ cells as live single cells, excluding KCs (Figure S8), the majority of which are recruited macrophages, and assessed the expression of the monocyte/macrophage markers Ly6C and CD11c. In mice consuming an ND, there were very few Ly6C- or CD11c-expressing cells among the F4/80^+^ cells, excluding KCs (Figure 5A). In contrast, the percentages and numbers of CD11c^+^/Ly6C^−^ and CD11c^−^/Ly6C^+^ cells were markedly increased by iHFC diet-feeding (Figure 5A,B). Between 8 and 24 weeks of iHFC diet-feeding, the number of CD11c^+^/Ly6C^−^ cells decreased, while the number of CD11c^−^/Ly6C^+^ cells did not significantly change (Figure 5B). Immunohistochemical analysis showed that CD11c^+^ and Ly6C^+^ cells accumulated in the liver during iHFC diet-feeding (Figure 5C,E). CD11c^+^ cells accumulated in a mesh-like pattern, surrounding the lipid droplets, thereby forming hCLSs (Figure 5D). In contrast, the Ly6C^+^ cells were small and accumulated in doughnut-like inflammatory lesions, with a lipid droplet in the center of each (Figure 5D). We also found that the percentage and cell number of dendritic cells (F4/80^−^/CD11b^+^/CD11c^+^/Ly6C^−^) were increased by iHFC diet-feeding (Figure S9). However, they were less than those of CD11c^+^ recruited macrophages (Figure S9). These results suggest that F4/80^+^ recruited macrophages consist of two different macrophage subsets, CD11c^+^/Ly6C^−^ and CD11c^−^/Ly6C^+^ cells, in the livers of iHFC-fed mice. Furthermore, their location in the liver suggests that these two macrophage subsets may have different roles in the development of NASH.

### 2.6. CD11c^+^ Cells Colocalize with Collagen Fibers in the Livers of iHFC-Fed Mice

**Figure 6:**
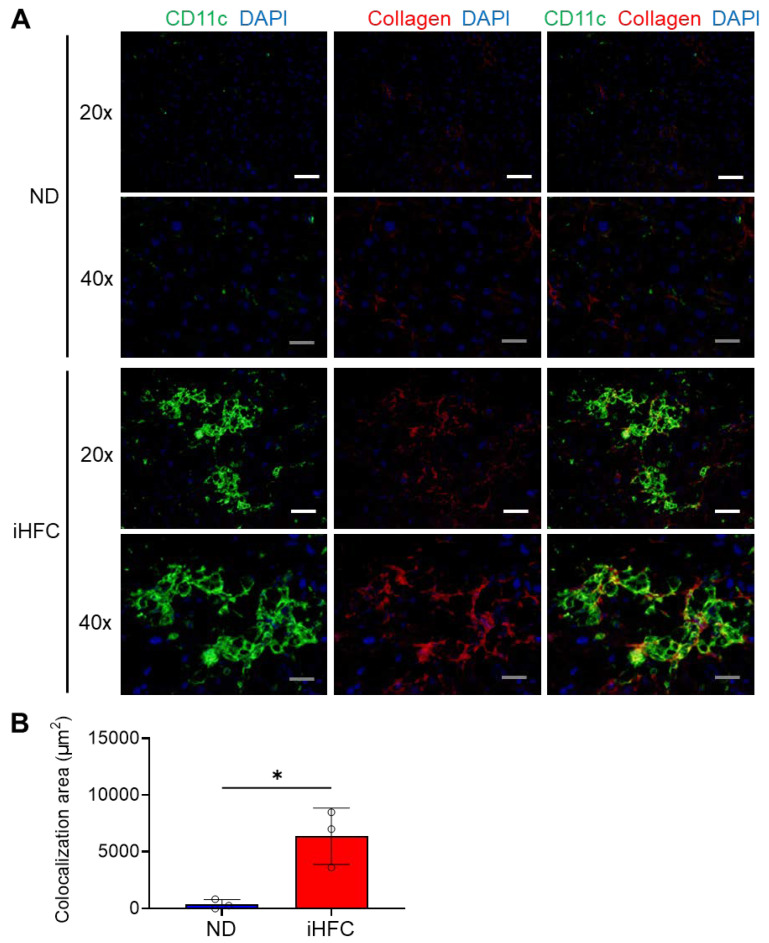
The iHFC diet accumulates CD11c^+^ cells which are colocalized with collagen fibers. (**A**) Representative histological images (20× or 40× magnification) of fluorescent immunohistochemistry for CD11c, collagen type 1, and DAPI of the livers from TSNO mice on the ND or iHFC diet for 12 weeks. White scale bars, 100 μm. Gray scale bars, 50 µm. (**B**) Colocalization areas were calculated as described in Figure S10 and the Materials and Methods. Data are shown as means ± SD. * *p* < 0.05. Statistical significance was evaluated by unpaired Student’s *t*-test.

Next, we performed immunofluorescence staining to clarify the role of CD11c^+^ recruited macrophages in the pathogenesis of liver fibrosis. Liver sections from mice fed the ND or iHFC diet for 12 weeks were analyzed, because Sirius red-stained collagen fibers and larger F4/80^+^ and CD11c^+^-areas were apparent at this time point (Figure 2A,C,E and Figure 5E). Mice consuming an ND showed very few CD11c^+^ cells and little collagen deposition (Figure 6A), but a number of CD11c^+^ cells had aggregated to form hCLSs in the livers of iHFC-fed mice (Figure 6A). Collagen deposition was evident in iHFC diet-fed mice (Figure 6A). Moreover, CD11c immunostaining significantly colocalized with collagen in the livers of the iHFC-fed mice (Figure 6A,B). These findings suggest that CD11c^+^ recruited macrophages in hCLSs promote advanced liver fibrosis in this model of NASH.

### 2.7. CD11c^+^/Ly6C^−^ and CD11c^−^/Ly6C^+^ Cells in the Liver Exhibit Distinct Gene Expression Profiles

**Figure 7:**
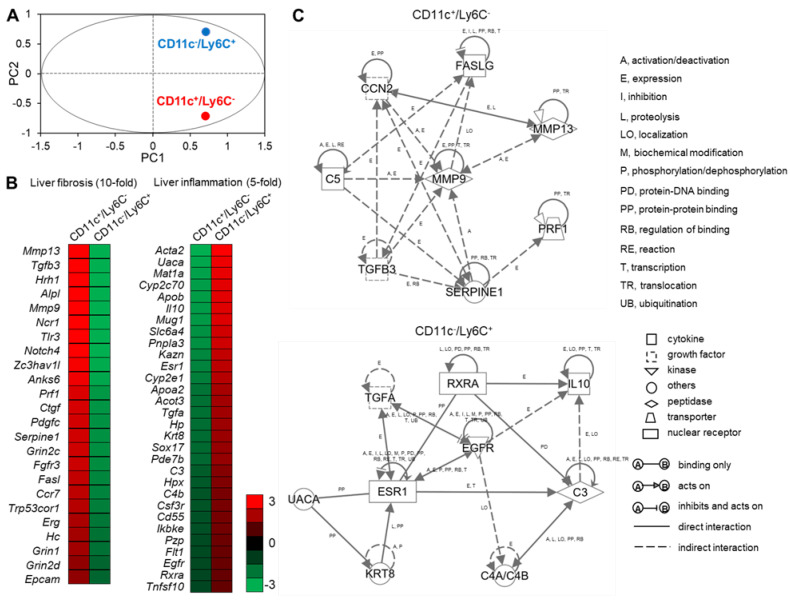
RNA sequence analysis of CD11c^+^/Ly6C^−^ and CD11c^−^/Ly6C^+^ cells in the liver from iHFC diet-fed TSNO mice. (**A**) PCA of two subsets of recruited macrophages from TSNO mice fed with the iHFC diet for 8 weeks. PCA was performed using Gene Spring. (**B**) Heatmap showing relative expression levels of upregulated or downregulated genes in two subsets of recruited macrophages from TSNO mice fed with the iHFC diet for 8 weeks. (**C**) The changes of the genes were analyzed using Ingenuity Pathways Analysis tools. The networks were displayed graphically as nodes (genes or proteins) and edges (the biological relationships between the nodes). Nodes and edges are displayed by various shapes and labels that represent the functional class of genes and the nature of the relationship between the nodes, respectively.

To elucidate the roles of CD11c^+^/Ly6C^−^ and CD11c^−^/Ly6C^+^ recruited macrophages in the pathogenesis of NASH, we performed RNA sequence analysis to compare the gene expression patterns of these subsets. Principal components analysis (PCA) showed that there were differences in the gene expression patterns of CD11c^+^/Ly6C^−^ and CD11c^−^/Ly6C^+^ cells (Figure 7A). We next performed a bioinformatic analysis to identify the molecular/cellular functions and gene networks of the differentially expressed genes in the two macrophage subsets. We focused on the gene ontology (GO) term “Inflammatory response”. Among these, we found a cluster of genes involved in liver fibrosis, with 24 genes showing 10-fold higher expression in CD11c^+^/Ly6C^−^ cells than in CD11c^−^/Ly6C^+^ cells (Figure 7B). These data are consistent with our findings that CD11c^+^ cells constitute hCLSs and colocalize with collagen fibers (Figure 5C,D and Figure 6). We also found a cluster of genes involved in liver inflammation, with 30 genes showing five-fold higher expression in CD11c^−^/Ly6C^+^ cells than in CD11c^+^/Ly6C^−^ cells (Figure 7B).

We also performed an analysis of the gene network associated with fibrosis in CD11c^+^/Ly6C^−^ cells, which included complement component 5 (C5), cellular communication network factor 2 (CCN2), Fas ligand (FASLG), matrix metallopeptidase 9 (MMP9), matrix metallopeptidase 13 (MMP13), perforin 1 (PRF1), serpin family E member 1 (SERPINE1), and transforming growth factor beta 3 (TGFB3) (Figure 7C, upper). Matrix metalloproteinases, including MMP9 and MMP13, play a key role in HSC activation in liver fibrogenesis [34,35]. CCN2 is also associated with fibrogenesis and HSC activation in the liver, and this gene is up-regulated by TGF-β1 stimulation [36,37]. Complement factor C5 has a role in murine biliary fibrogenesis and increases MMP9 expression [38]. A previous microarray study suggested that SERPINE1 and MMP9 might be involved in TGF-β-dependent fibrosis [39]. An analysis of PRF1-deficient mice demonstrated that PRF1 suppresses high-fat diet-induced NAFLD [40]. Fas, a member of the TNF receptor superfamily, contributes to mitochondrial dysfunction and the development of steatosis in response to a high-fat diet [41]. Furthermore, the concentration of soluble Fas and Fas ligand are high in children with NASH [42].

The analysis also revealed a gene network associated with liver inflammation in CD11c^−^/Ly6C^+^ cells, including complement component 3 (C3), complement component 4A (Rodgers blood group; C4A/C4B_2), epidermal growth factor receptor (EGFR), estrogen receptor 1 (ESR1), interleukin 10 (IL-10), keratin 8 (KRT8), retinoid X receptor alpha (RXRA), transforming growth factor alpha (TGFA), and uveal autoantigen with coiled-coil domains and ankyrin repeats (UACA) (Figure 7C, lower). IL-10 is an anti-inflammatory cytokine that has an antifibrotic effect by downregulating profibrogenic cytokines, such as TGF-β [43]. Furthermore, exogenous IL-10 can ameliorate CCl_4_-induced hepatic fibrosis in rats [44]. EGFR signaling in macrophages increases the expression of M2 macrophage-related cytokines, including IL-10 [45]. A RXR pan-agonist promotes the transcription of IL-10 [46]. The expression of EGFR and TGF-α, an EGFR ligand, in the liver correlates with the proliferation of normal and neoplastic hepatocytes [47]. EGFR signaling is required for estrogen-stimulated macrophage infiltration in the development of mammary tumorigenesis [48]. UACA is a proapoptotic protein that regulates NF-κB signaling [49], the expression of which is high in various cancers, including hepatocellular carcinoma [50]. KRT8 expression is high in patients with advanced liver fibrosis [51]. Macrophage sterol-responsive network (MSRN) proteins are coordinately regulated, and network dysregulation is important for foam cell formation and atherogenesis [52]. Of interest, C3 is an MSRN protein and aortic atherosclerosis is worse in aortas from C3-deficient mice than in controls [53]. The complement anaphylatoxin C4a inhibits the C3a-induced leukocyte reaction [54].

These findings suggest that CD11c^+^/Ly6C^−^ cells might promote liver fibrosis and HSC activation, whereas CD11c^−^/Ly6C^+^ cells might play an anti-inflammatory role and promote tissue repair, similar to M2 macrophages. We also found that CD11c^+^/Ly6C^−^ and CD11c^−^/Ly6C^+^ cells express a variety of chemokine receptors, and their expression patterns differ between the two macrophage subsets (Figure S11). This data suggests that chemokine receptors might play important roles in the infiltration of these two macrophage subsets into the liver.

## 3. Discussion

In the present study, we characterized the macrophage subsets present in the liver of a newly established mouse model of NASH with advanced fibrosis. The objectives of the study were to (1) characterize the changes in macrophage subsets during the development of NASH, (2) define the phenotypes of these macrophage subsets, and (3) assess their roles in the progression of inflammation and fibrosis in the model. We have shown that two distinct macrophage subsets, CD11c^+^/Ly6C^−^ and CD11c^−^/Ly6C^+^ cells, accumulate in the liver during the development of NASH in these mice. Furthermore, our data suggest that CD11c^+^/Ly6C^−^ cells, which constitute hCLSs, contribute to the development of liver fibrosis. In addition, we have identified evidence that CD11c^−^/Ly6C^+^ cells, which differ in their localization in the liver from CD11c^+^/Ly6C^−^ cells, are implicated in the pathogenesis of anti-inflammation/tissue repair in NASH. Their specific roles require further investigation, but the present findings provide novel insight into the importance of liver macrophages in the initiation of inflammation and fibrosis in NASH.

Liver fibrosis is a feature of NASH, which can progress to liver cirrhosis. Ichimura-Shimizu et al. established a novel mouse model of diet-induced NASH using TSNO mice [32]. It was shown that iHFC diet-fed TSNO mice exhibit advanced liver fibrosis that mimics that of human NASH [32]. The origin, cell surface markers, and biological function of resident and recruited macrophages have been characterized in recent years, but further understanding of the roles of these macrophage subsets in the development of hepatic inflammation and fibrosis is critical to evaluate their potential as therapeutic targets. Therefore, we used this model as a tool to gain insight into the biological functions of macrophages in the development of NASH, and in particular with regard to advanced fibrosis. We found that the number and percentage of F4/80^Hi^/CD11b^Int^ KCs decreased during the development of NASH induced by iHFC diet-feeding, while those of F4/80^Int^/CD11b^Int-Hi^ recruited macrophages markedly increased (Figure 4B,C). Furthermore, the composition of the resident KCs was altered, with TIM4-negative cells, which are of monocyte origin, increasing in abundance in the F4/80^Hi^ KC population (Figure S6A and S6B). TIM4-negative monocyte-derived cells accumulate in the liver and take on the majority of features of KCs during the development of NASH in mice [21]. These findings are consistent with the results of previous studies of the methionine/choline deficiency model of NASH and the high-fat, high-sucrose diet model of NAFLD/NASH [21,55]. Therefore, the iHFC model is not atypical and the results generated can be compared with those obtained using conventional models of NASH.

Recruited monocyte-derived macrophages populate the resident KC niche in the livers of patients with NASH [56]. In addition to TIM4-negative KCs, two subsets of monocyte-derived macrophage have previously been described. Lipid-associated macrophages (LAMs) were first described in obese adipose tissue and express high levels of *Cd9* and *Trem2* [57]. Their counterparts in the liver, hepatic LAMs, express high levels of osteopontin, a biomarker of NASH, and are associated with the development of fibrosis [56]. LAMs contain a subset characterized by the expression of *Cx3cr1/Ccr2* and *Trem2* [21]. This subset is referred to as C-LAMs and forms hCLSs in regions of HSC expansion [21,56]. C-LAMs show higher expression of CD11c than Cx3cr1^lo^ macrophages and TIM-negative monocyte-derived KCs [21,58]. F4/80^Int^/CD11b^Int-Hi^ recruited macrophages include CD11c^+^ cells, which are markedly increased in number by iHFC diet-feeding (Figure 5A–C). In addition, CD11c^+^/Ly6C^−^ cells express various chemokine receptors, including *Cx3cr1* and *Ccr2* genes (Figure S11). Thus, CD11c^+^/Ly6C^−^ cells correspond to C-LAMs in our NASH model. In the present study, we analyzed the macrophage subsets at various time points during the feeding period, and found that CD11c^+^/Ly6C^−^ cells had increased in number by the 4-week time point. Therefore, this recruited macrophage subset might contribute to the formation of hCLSs and the development of advanced fibrosis from an early stage of iHFC diet-feeding.

The presence of hCLSs is associated with liver fibrosis in NASH in both mice and humans [8]. Here, we have shown that hCLS formation is induced by iHFC diet-feeding (Figure 4D). Furthermore, the hCLSs were composed of CD11c^+^ cells, which colocalized with collagen fibers in the livers of iHFC-fed mice (Figure 5C,D and Figure 6). Thus, CD11c^+^/Ly6C^−^ cells are a key macrophage subset in hCLSs. However, it is controversial whether hCLS formation is beneficial or detrimental for tissue remodeling in NASH. It has been shown previously that macrophage accumulation, especially that induced by CCR2 signaling, is important for hCLS formation and the development of fibrosis [21,59]. In addition, pharmacological inhibition of CCR2 has been shown to reduce liver fibrosis in NASH [59], whereas CCR2 deficiency reduces macrophage aggregation and increases fibrosis [17,21]. These results suggest that the timing of CCR2 inhibition might affect the composition of the tissue macrophage population and tissue remodeling. Future studies should determine whether inhibition of CCR2 affects the development of advanced fibrosis in this model of NASH.

RNA sequence analysis revealed some further aspects of the function of the two macrophage subsets: CD11c^+^/Ly6C^−^ and CD11c^−^/Ly6C^+^ cells. These subsets have distinct gene expression profiles (Figure 7A,B), and gene network analysis indicated that CD11c^+^/Ly6C^−^ cells promote liver fibrosis and HSC activation, whereas CD11c^−^/Ly6C^+^ cells play an anti-inflammatory role and promote tissue repair, similar to M2 macrophages (Figure 7C). Our findings regarding CD11c^+^ recruited macrophages are largely consistent with those of previous studies with respect to their histological distribution and biological function [21,56]. “Restorative macrophages”, which are characterized by Ly6C^lo^ expression in mice, that are involved in the resolution of fibrosis and accumulate during the restorative phase following tissue damage in mice with CCl_4_-induced NASH have been identified [60]. This macrophage subset differentiates from infiltrating Ly6C^Hi^ monocyte/macrophages, which undergo a functional switch in the course of liver injury [60]. These data are consistent with our findings that CD11c^−^/Ly6C^+^ macrophages are anti-inflammatory and assist with tissue repair, and suggest that CD11c^−^/Ly6C^+^ macrophages might differentiate from Ly6C^+^ monocytes. However, it has been reported that the administration of a CCL2 inhibitor inhibits the infiltration of Ly6C^+^ monocytes into the liver and their differentiation into Ly6C^+^ macrophages, thereby reducing fibrosis and inflammation [61]. These results suggest that the effects of Ly6C^+^ macrophages might differ in the various models of NASH and on the basis of the phase of the disease. Future studies should investigate the dynamics of CD11c^−^/Ly6C^+^ macrophages and their interactions with other macrophage subsets and HSCs during various phases in the model of NASH used.

In conclusion, we have provided novel insights into the recruited macrophage subsets that characterize NASH and their roles in the development of liver inflammation and advanced fibrosis. Although the details of their origins and biological functions require more investigation, the present findings provide information that should aid the development of therapeutic agents for NASH that target macrophages.

## 4. Materials and Methods

### 4.1. Mice

Male TSNO mice (6-weeks old) were purchased from the Institute of Animal Reproduction (Ibaraki, Japan) and maintained in microisolator cages under specific pathogen-free conditions in the animal facility of Toyama Prefectural University under standard light conditions (12-h light/dark cycle) and were allowed free access to water and food. Male 7-week old TSNO mice were fed ad libitum either a normal diet (ND) (MF, Oriental-Yeast, Tokyo, Japan) or an iHFC diet, which is high in fat, cholesterol, and cholate (69.5% standard chow, 28.75% palm oil, 1.25% cholesterol, and 0.5% cholate) (Hayashi Kasei, Osaka, Japan) for the indicated period. The animal care policies and procedures/protocol used in the experiments were approved by the Animal Experiment Ethics Committee of Toyama Prefectural University (Approved No. R1-2 and R3-6).

### 4.2. Plasma Biochemical Analysis

Blood samples were collected from the inferior vena cava, and plasma samples were also collected. Plasma levels of alanine aminotransferase (ALT), aspartate transaminase (AST), total cholesterol (T-CHO), and triglyceride (TG) were measured by DRI-CHEM NX700 (Fujifilm, Tokyo, Japan), following the manufacturer’s instruction.

### 4.3. Isolation of Non-parenchymal Cells from the Liver

To isolate non-parenchymal cells from the liver, mice were placed under anesthesia (isoflurane) and perfusion was performed with PBS. Isolation of non-parenchymal cells was performed by the Liver Dissociation Kit (Miltenyi Biotech, Bergisch Gladbach, Germany), following the manufacturer’s instruction. Then, the cell suspension was passed through a cell strainer (100 μm) and used for flow cytometry analysis and cell sorting.

### 4.4. Flow Cytometry Analysis

The non-parenchymal cells (2 × 10^5^) were incubated with anti-mouse FcγR (2.4G2) to block binding of the fluorescence-labeled antibodies to FcγR. After 20 min, the cells were stained with predetermined optimal concentrations of the respective antibodies. Then, 7-amino-actinomycin D (7-AAD) (BD Biosciences, San Diego, CA, USA) was used to exclude dead cells. Flow cytometry analyses were conducted on a FACSCantoII (Becton Dickinson & Co., Mountain View, CA, USA), and the data were analyzed with Flowjo software (BD Biosciences). The antibodies for flow cytometry were listed in Supplementary Table S1.

### 4.5. Cell Sorting

For sorting of CD45^+^ or CD45^−^, non-parenchymal cells were stained with APC-conjugated anti-CD45. For sorting of Ly6C^+^/CD11c^−^ and Ly6C^−^/CD11c^+^ cells from non-parenchymal cells, harvested cells were stained with FITC-conjugated anti-Ly6C, PE-conjugated anti-CD11c, and APC-conjugated anti-CD45. The antibodies for cell sorting were listed in Supplementary Table S1. Cells were sorted on a FACSMelody (Becton Dickinson & Co.). Sorting gates are presented in Supplementary Figure S12.

### 4.6. Preparation of RNA and cDNA

Total RNA was extracted using NucleoSpin RNA Mini kit (Macherey-Nagel, Düren, Germany) and TRIzol^®^ LS Reagent (Invitrogen, Carlsbad, CA, USA), following the manufacturer’s instructions. RNA was reverse transcribed with a PrimeScript^®^ RT regent kit (Takara Bio Inc., Shiga, Japan), following the manufacturer’s instructions.

### 4.7. Quantitative Real-Time PCR

qRT-PCR was performed with a FastStart Universal Probe Master (Roche Applied Science, Mannheim, Germany) and analyzed with a CFX96 Touch™ Real-Time PCR Detection System (Bio-Rad, Hercules, CA, USA), following the manufacturer’s instructions. Relative transcript abundance was normalized for that of Hprt mRNA. The information for the TaqMan primer/probe (Applied Biosystems, Carlsbad, CA, USA) used for real-time PCR is listed in Supplementary Table S2.

### 4.8. Western Blotting Analysis

Liver tissue was lysed using RIPA buffer and mechanically disrupted using a Minilys homogenizer (Bertin Instrument, Montigny-le-Bretonneux, France) with three rounds of mechanical disruption (bead-beating at 5000 rpm for 1 min). The samples were centrifuged, and the resulting supernatants were each mixed with SDS-PAGE sample buffer (ATTO, Tokyo, Japan), subjected to polyacrylamide gel electrophoresis, and transferred to PVDF membranes. PVDF membranes with proteins were incubated with the following primary antibodies; anti-p38 (Cell Signaling, Danvers, MA, USA), anti-phosphorylated p38 (Cell Signaling). Secondary antibody HRP-conjugated anti-rabbit IgGs was purchased from Cell Signaling. Protein expression levels were visualized by Immobilon Crescendo Western HRP Substrate (Millipore, Billerica, MA, USA), and images were captured using a LAS-4000 luminescent image analyzer (Fujifilm).

### 4.9. Histological and Immunohistochemistry Analysis

Portions of the liver were excised and fixed immediately with 4% formaldehyde at room temperature. Paraffin-embedded tissue sections were cut into 4-μm slices and placed on slides. Sections were stained with hematoxylin and eosin or Sirius red, according to standard procedures. Anti-F4/80, anti-CD11c, and anti-Ly6C antibodies were purchased from Cedarlane Laboratories (Ontario, Canada), Invitrogen, and Abcam (Cambridge, MA, USA), respectively. Positive areas for F4/80 and Sirius red were measured using ImageJ software [62]. Histologic steatosis, lobular inflammation, and hepatocyte ballooning were assessed according to the criteria proposed by Kleiner et al. [11]. All histological analyses were performed by pathologists (K.Tsuneyama and M.I.-S.), and the histological scores and grade were determined in a blinded manner.

### 4.10. Fluorescent Immunohistochemistry Analysis

Next, 7-μm frozen sections were incubated with anti-CD11c (Invitrogen) and secondary antibody (anti-hamster IgG, Southern Biotech, Birmingham, AL, USA), and sequentially incubated with TSA Fluorescein System (Akoya Biosciences, Marlborough, MA, USA). The sections were also incubated with anti-collagen type 1 (Novotec, Bron, France) and secondary antibody (anti-rabbit IgG Alexa Fluor 594, Abcam). Finally, the sections were incubated with DAPI (Invitrogen). Images were acquired using a BX50 microscope and its imaging system (Olympus, Tokyo, Japan). The positive signal of each colocalized area was selected according to the method of Tolivia et al. (Figure S10) [63] and analyzed using Adobe Photoshop CS software, version 8.

### 4.11. RNA Sequence

cDNA synthesis and amplification from 1 ng total RNA were performed by using the SMART-Seq HT kit (Takara Bio Inc.), followed by the tagmentation using the Nextera XT DNA library prep kit (Illumina, San Diego, CA, USA). Nextera PCR Master Mix (Illumina) and Nextera-DNB Conversion Primer (Supplementary Table S3) were used for PCR amplification of the tagmented cDNA. Sequence libraries with index sequences were constructed from the amplified cDNA by using Prime STAR HS DNA polymerase (Takara Bio Inc.) and index primer sets (Supplementary Table S3). The libraries were subjected to cluster generation and paired-end sequencing analysis (2 × 200 bp) with the DNBSEQ (MGI, Wuhan, China). These steps following total RNA extraction were performed by Bioengineering Lab Co., Ltd. (Sagamihara, Japan).

Trimming of the low-quality reads and sequence adaptor (NNTCGTCGGCAGCGTCAGATGTGTATAAGAGACAG or NNGTCTCGTGGGCTCGGAGATGTGTATAAGAGACAG) from raw sequence reads were performed by using Trim Galore Ver 0.6.6, followed by the mapping of the processed sequenced reads to the mouse genome (mm10, RefSeq) with HISAT2 Ver 2.1.0. Samtools Ver 1.9 were used for the conversion of the obtained SAM files to BAM format followed by gene expression profiling by using Strand NGS software Ver4.0. For global normalization, raw sequence read counts were processed using Trimmed means of M value (TMM) algorithm. Principal component analysis (PCA) was performed using R Version 3.6.0. Gene network analysis was performed using Ingenuity Pathway Analysis tools (Ingenuity Systems, Mountain View, CA), following the extraction of genes differentially expressed by >5.0- or >10.0-fold.

### 4.12. Statistical Analysis

Statistical significance was evaluated by two-way ANOVA followed by post hoc Sidak test for multiple comparison, and Student’s *t*-test for two groups comparison. When we analyzed Student’s *t*-tests, we first performed an F-test on GraphPad Prism 9 software (GraphPad; San Diego, CA, USA) to confirm that the *p*-value of the F test was >0.05 and equally distributed. Statistical analysis was performed using GraphPad Prism 9 software. *p* < 0.05 was considered statistically significant. The results were presented as the mean ± SD.

## Data Availability

The data that support the findings of this study are available from the corresponding author upon reasonable request.

## Supplementary Materials

The following supporting information can be downloaded at: https://www.mdpi.com/article/10.3390/ijms232113251/s1.
