# Supplementary material for: Roles of Macrophages in Advanced Liver Fibrosis, Identified Using a Newly Established Mouse Model of Diet-Induced Non-Alcoholic Steatohepatitis
Source: Int J Mol Sci. 2022 Oct 31;23(21):13251. doi: 10.3390/ijms232113251 (PMC9654696; doi:10.3390/ijms232113251)
Supplement: Supplementary file 1 [file ijms-23-13251-s001.zip › ijms-1985598-supplementary.pdf]

Antibody	Clone	Conjugate	Source
Anti-CD11c	HL3	PE	BD Pharmingen
Anti-Ly6G/Gr-1	RB6-8C5	PE	BD Pharmingen
Anti-CD11b/Mac-1	M1/70	APC-Cy7	BD Pharmingen
Anti-CD45	30-F11	APC	BioLegend
Anti-F4/80	BM8	PE	BioLegend
Anti-F4/80	BM8	FITC	BioLegend
Anti-TIM4	RMT4-54	PE	BioLegend
Anti-Ly6C	HK1.4	FITC	BioLegend

Supplementary Table S1. Antibodies for flow cytometry.

The antibodies for flow cytometry and cell sorting are listed. The antibodies were purchased from BD Pharmingen (San Diego, CA) or BioLegend (San Diego, CA).

Gene	Gene Symbol	Gene Name	Assay ID
Hprt	Hprt1	hypoxanthine guanine phosphoribosyl transferase	Mm00446968_m1
TNF-a	Tnf	tumor necrosis factor	Mm00443258_m1
MCP-1	Ccl2	chemokine (C-C motif) ligand 2	Mm00441243_g1
iNOS	Nos2	nitric oxide synthase 2, inducible	Mm01309898_m1
F4/80	Adgre1	adhesion G protein-coupled receptor E1	Mm00802530_m1
CD11c	Itgax	integrin alpha X	Mm00498698_m1
Tgfb-1	Tgfb1	transforming growth factor, beta 1	Mm01178820_m1
TIMP-1	Timp1	tissue inhibitor of metalloproteinase 1	Mm00441818_m1
Col1a-1	Col1a1	collagen, type 1, alpha 1	Mm00801666_g1
a-SMA	Acta2	actin, alpha 2, smooth muscle, aorta	Mm00725412_s1
Desmin	Des	desmin	Mm00802455_m1
IL-1beta	Il1b	interleukin 1 beta	Mm01336189_m1

Supplementary Table S2. Primers for RT-qPCR.

The primers were purchased from Applied Biosystems (Waltham, MA).

**Nextera-DNB Conversion Primer**	
Forward	GAACGACATGGCTACGATCCGACTTNNTCGTCGGCAGCGTC
Reverse	GTCTTCCTAAGACCGCTTGGCCTCCGACTTNNGTCTCGTGGGCTCGG
**Index primer sets**	
Forward	GAACGACATGGCTACGATCCGAC
Reverse	TGTGAGCCAAGGAGTTG [index] TTGTCTTCCTAAGACCGCTTGGCC

Supplementary Table S3. Primer sequences for RNA sequence analysis.

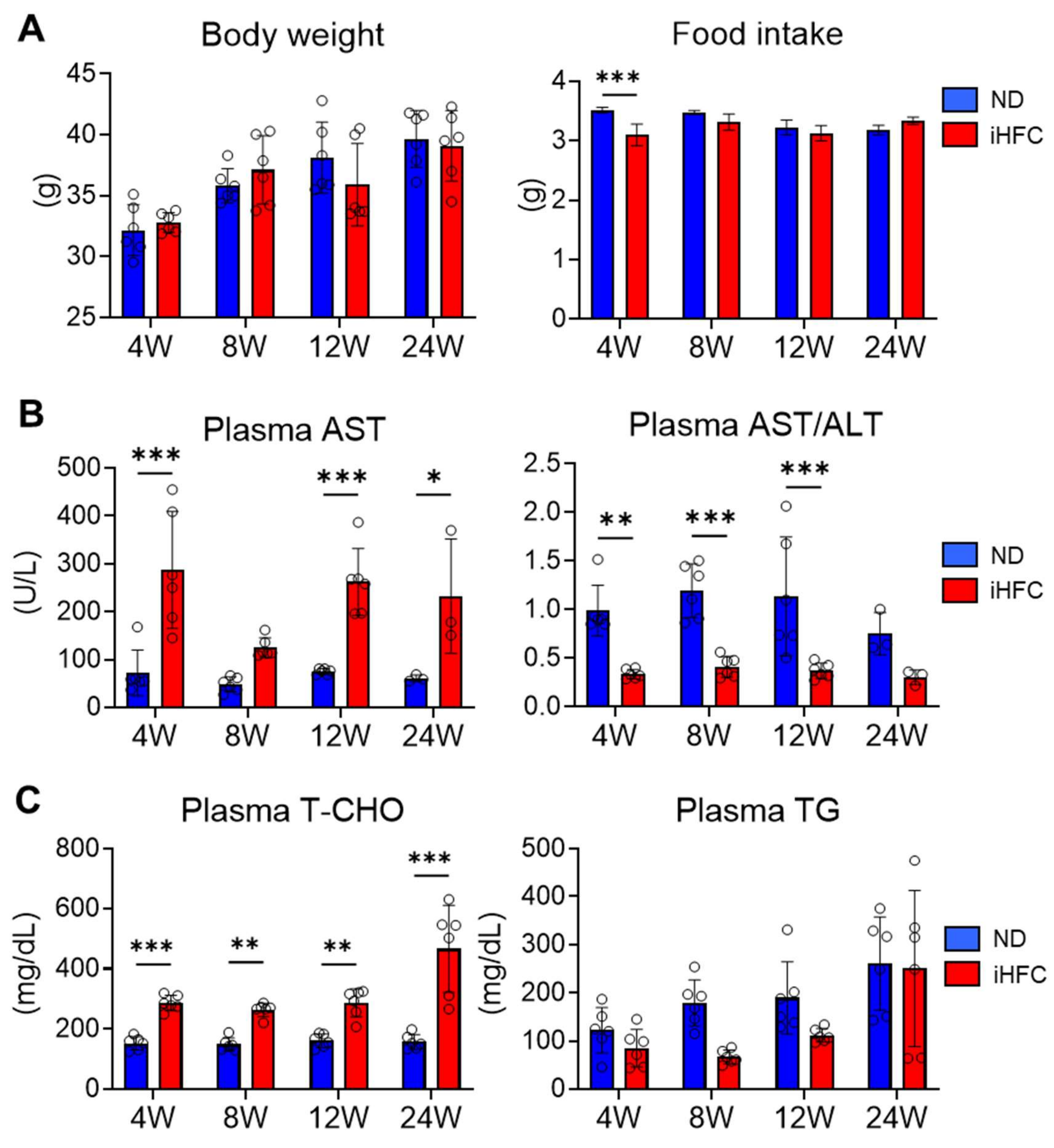

Supplementary Figure S1. Body weight, food intake, and plasma AST, T-CHO, and TG levels of TSNO mice on the ND or iHFC diet.

(A) Body weights and daily food intakes were measured for TSNO mice fed with the ND or iHFC diet for the indicated time periods (n = 6 per group). (B) Plasma AST levels were measured for TSNO mice fed with the ND or iHFC diet for the indicated time periods (n=3-6 per group). The ratio of AST to ALT in plasma was also calculated. (C) Plasma T-CHO and TG levels were measured for TSNO mice fed with the ND or iHFC diet for the indicated time periods (n=6 per group). Data are shown as means ± SD. $^{*}P < 0.05$, $^{**}P < 0.01$, $^{***}P < 0.001$. Statistical significance was evaluated by 2-way ANOVA followed by post-hoc Sidak test.

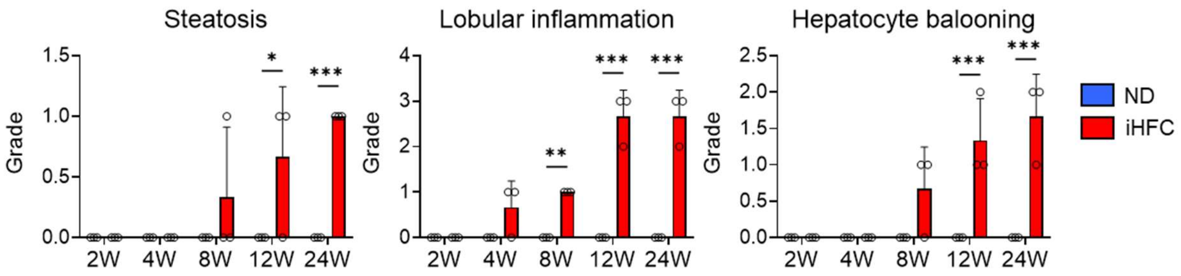

Supplementary Figure S2. Grade and stage of histologic steatosis, lobular inflammation, and ballooning hepatocytes of the liver from TSNO mice.

Steatosis (0 to 3), lobular inflammation (0 to 3), and hepatocyte ballooning (0 to 2) were assessed according to the criteria proposed by Kleiner et al. as described in Materials and Methods (n = 3 per group). Data are shown as means ± SD. $^{*}P < 0.05$, $^{**}P < 0.01$, $^{***}P < 0.001$. Statistical significance was evaluated by 2-way ANOVA followed by post-hoc Sidak test.

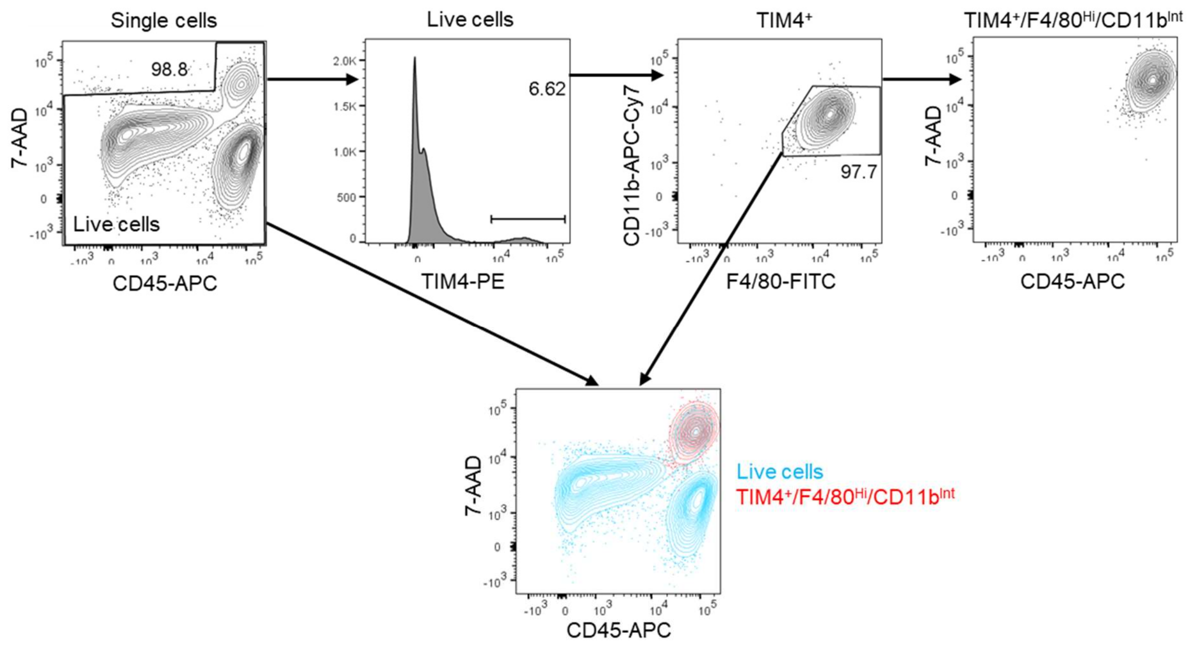

Supplementary Figure S3. Representative flow cytometry data of CD45, TIM4, F4/80, and CD11b expressions on live non-parenchymal cells of the livers from TSNO mice. Single non-parenchymal cells of the livers from ND-fed TSNO mice contained highly auto-fluorescent $CD45^+$ cells. Staining of these cells with an antibody to TIM4, a specific marker of KCs, revealed that $TIM4^+$ KCs were $F4/80^{Hi}/CD11b^{Int}$ and highly auto-fluorescent $CD45^+$ cells.

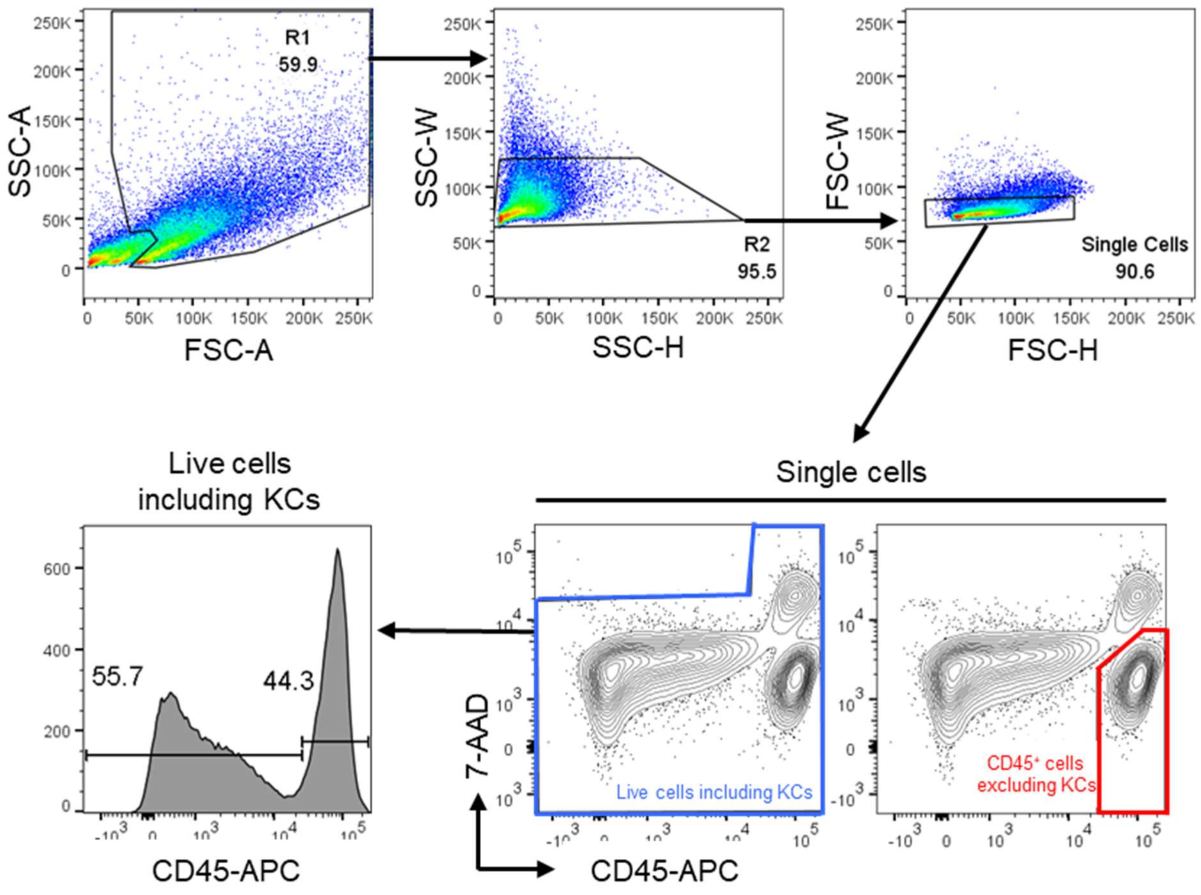

Supplementary Figure S4. Gating strategy for flow cytometry analysis of non-parenchymal cell of the liver from TSNO mice.

Since KCs were highly auto-fluorescent CD45$^+$ cells (Figure S3), we used two different gating strategies to analyze CD45$^+$ cells, depending on whether the KCs were being assessed or not. To examine CD45$^+$ cells including KCs, single cells were first analyzed with a plot of CD45 and 7-AAD and gated on live cells including highly fluorescent CD45$^+$ cells (blue gate), followed by a histogram of CD45. To examine CD45$^+$ cells excluding KCs, single cells were gated on CD45 expressing cells excluding highly fluorescent CD45$^+$ cells (red gate).

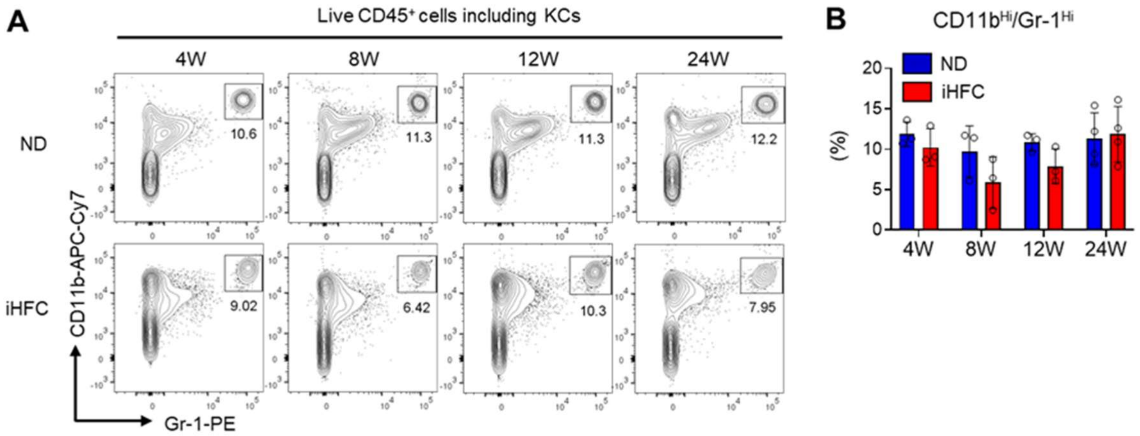

Supplementary Figure S5. Neutrophils are in the liver from TSNO mice without any change in their percentages between ND and iHFC diet-fed mice.

(A) Representative flow cytometry data of CD11b and Gr-1 expression on $CD45^+$ non-parenchymal cells of the livers from TSNO mice on the ND or iHFC diet for the indicated time periods. (B) Percentage of neutrophils ($CD11b^{Hi}/Gr\text{-}1^{Hi}$) was determined by flow cytometry analysis done in Figure S5A (n = 3 or 4 per group). Data are shown as means ± SD.

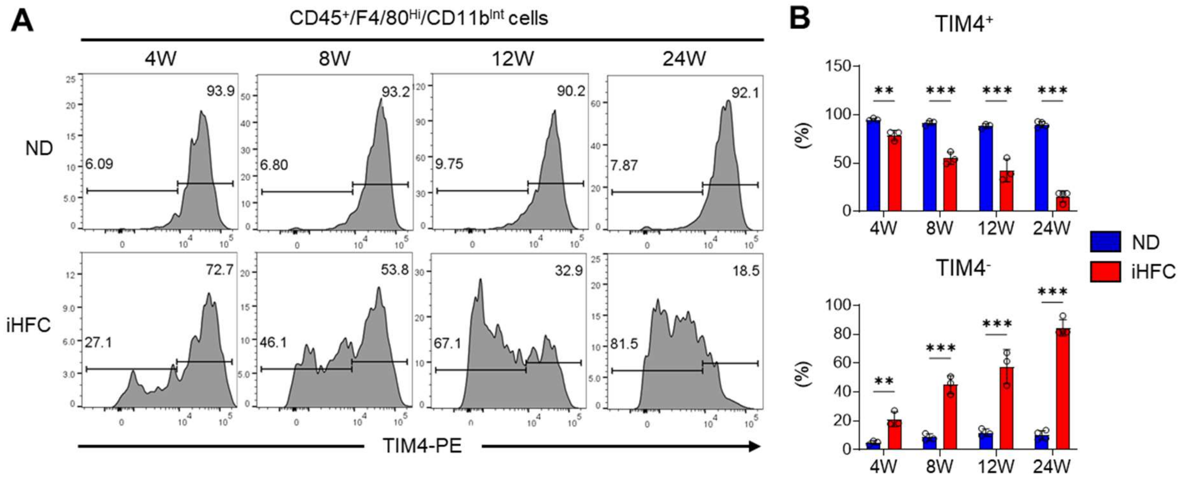

Supplementary Figure S6. TIM4 expression on CD45$^+$/F4/80^{Hi}/CD11b^{Int} KCs is dramatically decreased with feeding period of iHFC.

(A) Representative flow cytometry data of TIM4 expression on CD45$^+$/F4/80^{Hi}/CD11b^{Int} KCs of the livers from TSNO mice on the ND or iHFC diet for the indicated time periods. (B) Percentages of TIM4$^+$ and TIM4$^-$ cells in CD45$^+$/F4/80^{Hi}/CD11b^{Int} KCs were determined by flow cytometry analysis done in Figure S6A (n = 3 or 4 per group). Data are shown as means ± SD. $^{**}P < 0.01$, $^{***}P < 0.001$. Statistical significance was evaluated by 2-way ANOVA followed by post-hoc Sidak test.

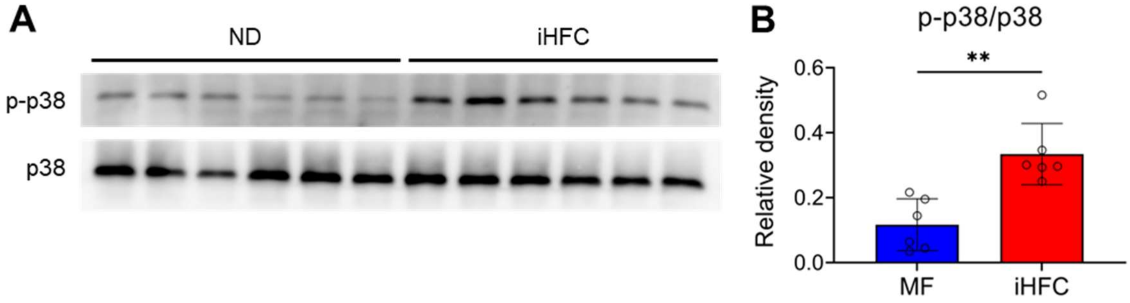

Supplementary Figure S7. iHFC diet induces the phosphorylation of p38 in the livers from TSNO mice.

(A)Western blotting analysis of p38 and phosphorylated p38 (p-p38) in the livers from TSNO mice fed with the ND or iHFC diet for 4 weeks (n = 6 per group). (B) Relative density for p-p38/p38 was measured using ImageJ software based on the western blotting analysis data in Figure S7A. Data are shown as means ± SD. $^{**}P < 0.01$. Statistical significance was evaluated by unpaired Student's t-test.

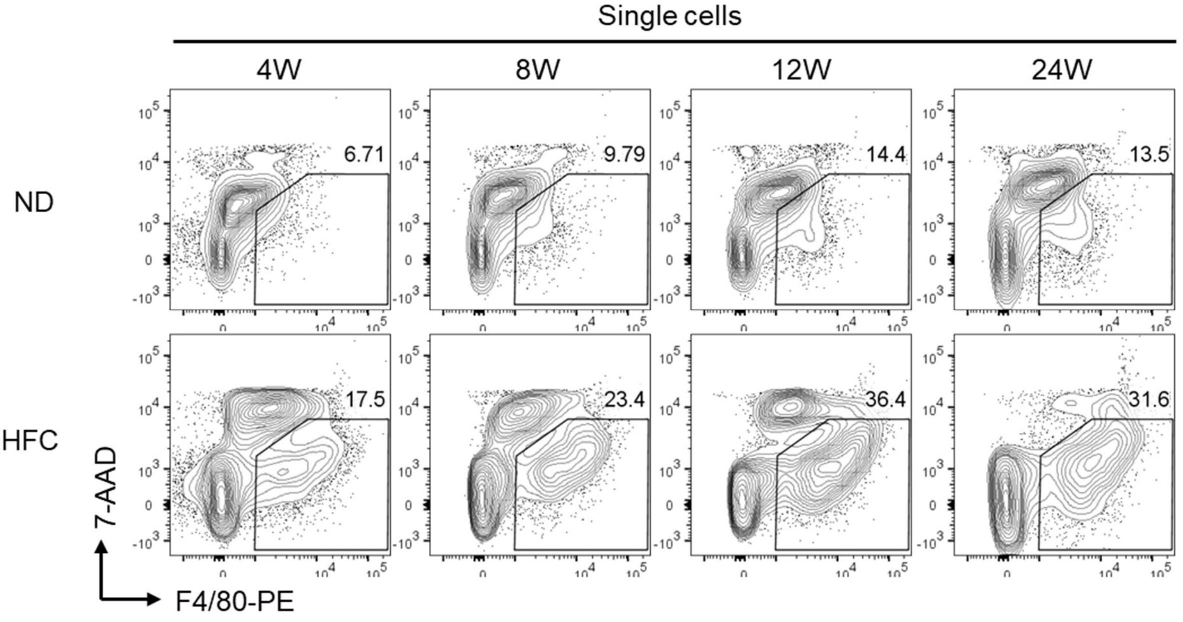

Supplementary Figure S8. Gating strategy for flow cytometry analysis of F4/80$^+$ non-parenchymal cell, excluding KCs, of the liver from TSNO mice.

Non-parenchymal cells were isolated from the liver from ND- or iHFC diet-fed TSNO mice for the indicated weeks. Single non-parenchymal cells were analyzed with a plot of F4/80 and 7-AAD and gated on live F4/80$^+$ cells excluding dead cells and highly fluorescent F4/80-positive KCs. Then, we examined the expression of Ly6C and CD11c on F4/80$^+$ recruited macrophages, as shown in Figure 5A.

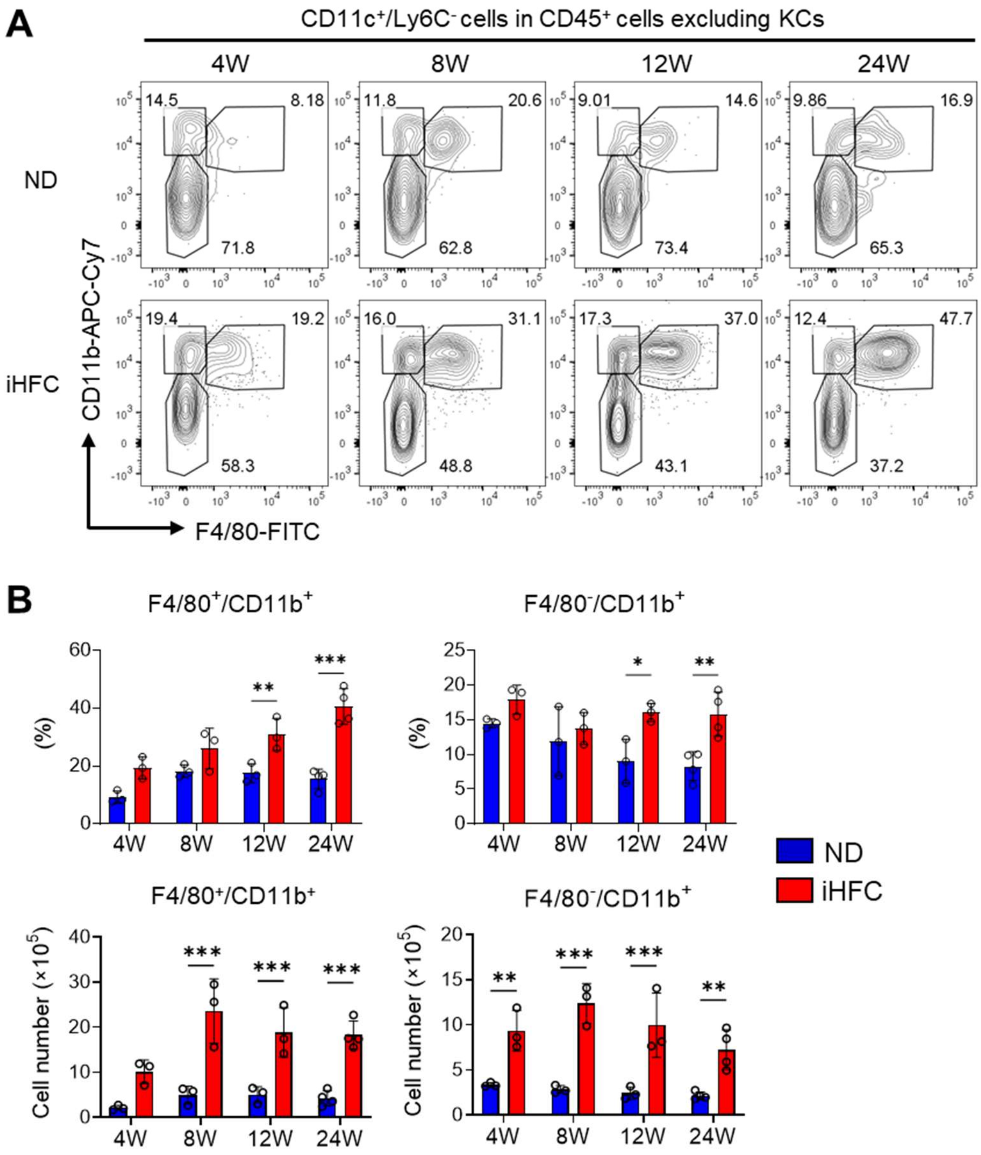

Supplementary Figure S9. Flow cytometry analysis of F4/80⁻/CD11b⁺/CD11c⁺/Ly6C⁻dendritic cells in the liver from TSNO mice.

(A) Representative flow cytometry data of F4/80 and CD11b expression on $CD45^+/CD11c^+/Ly6C^-$ cells of the livers from TSNO mice on the ND or iHFC diet for the indicated time periods. (B) Percentage of $CD11c^+$ recruited macrophages ($F4/80^+/CD11b^+$) and dendritic cells ($F4/80^-/CD11b^+$) in $CD45^+/CD11c^+/Ly6C^-$ cells was determined by flow cytometry analysis done in Figure S9A (n = 3 or 4 per group). Cell number of these cells was also calculated. Data are shown as means ± SD. $^*P < 0.05$, $^{**}P < 0.01$, $^{***}P < 0.001$. Statistical significance was evaluated by 2-way ANOVA followed by post-hoc Sidak test.

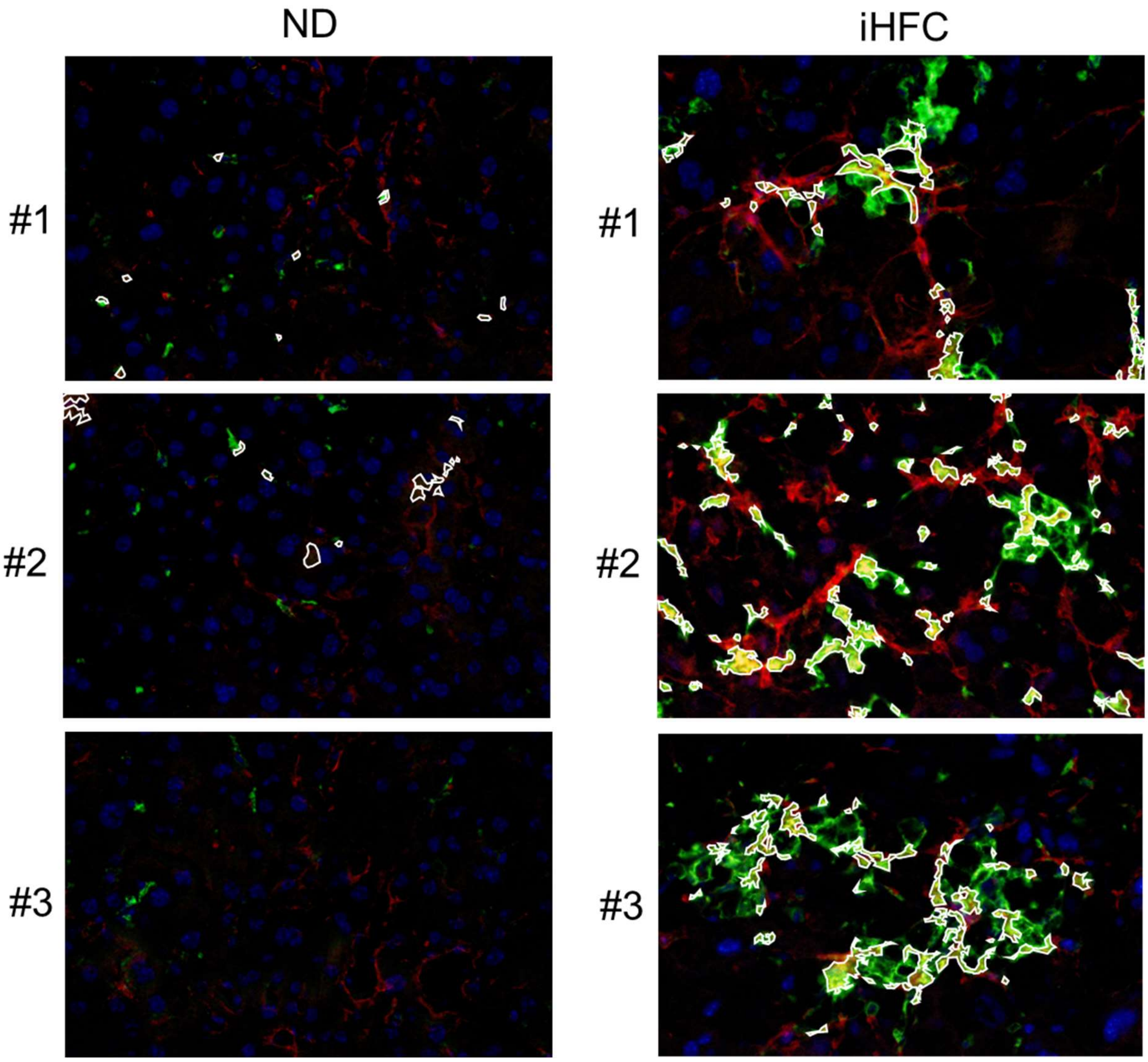

Supplementary Figure S10. Colocalized areas of CD11c and collagen immunostainings in the liver from ND or iHFC diet-fed TSNO mice.

Representative histological images (40x magnification) of fluorescent immunohisto-chemistry for CD11c, collagen type 1, and DAPI of the livers from TSNO mice on the ND or iHFC diet for 12 weeks (3 mice/group). The positive signal of each colocalized area was selected according to the method of Tolivia J et al. (ref. 63).

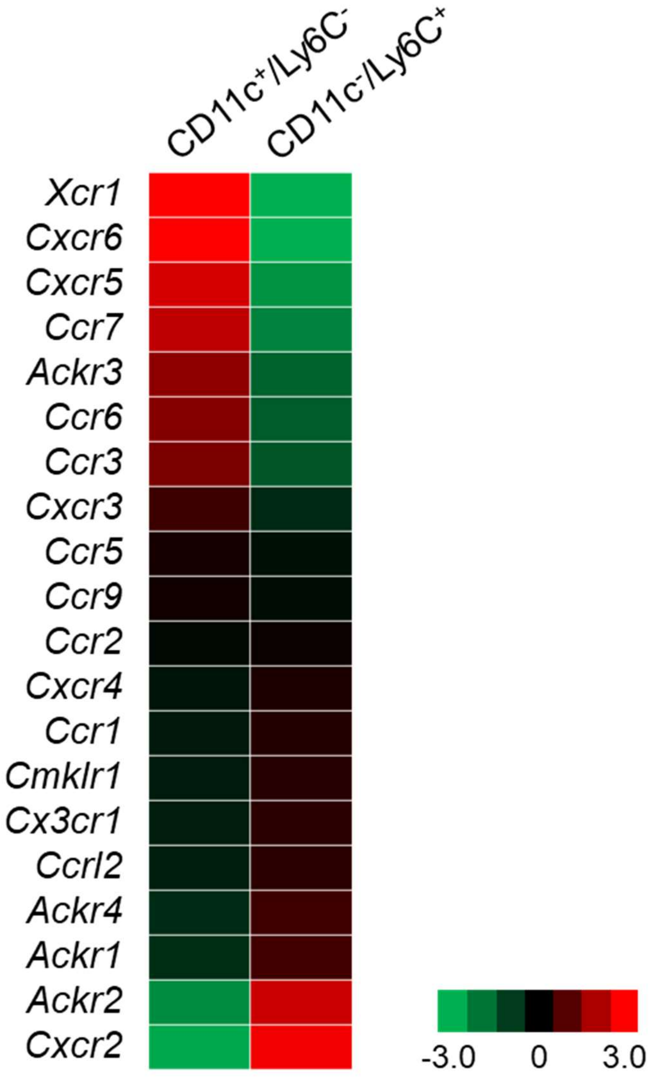

Supplementary Figure S11. RNA sequence analysis of CD11c^{+}/Ly6C^{-} and CD11c^{-}/ Ly6C^{+} cells in the liver from iHFC diet-fed TSNO mice.

Heatmap showing relative expression levels of chemokine receptor-related genes in two subsets of recruited macrophages from TSNO mice fed with the iHFC diet for 8 weeks.

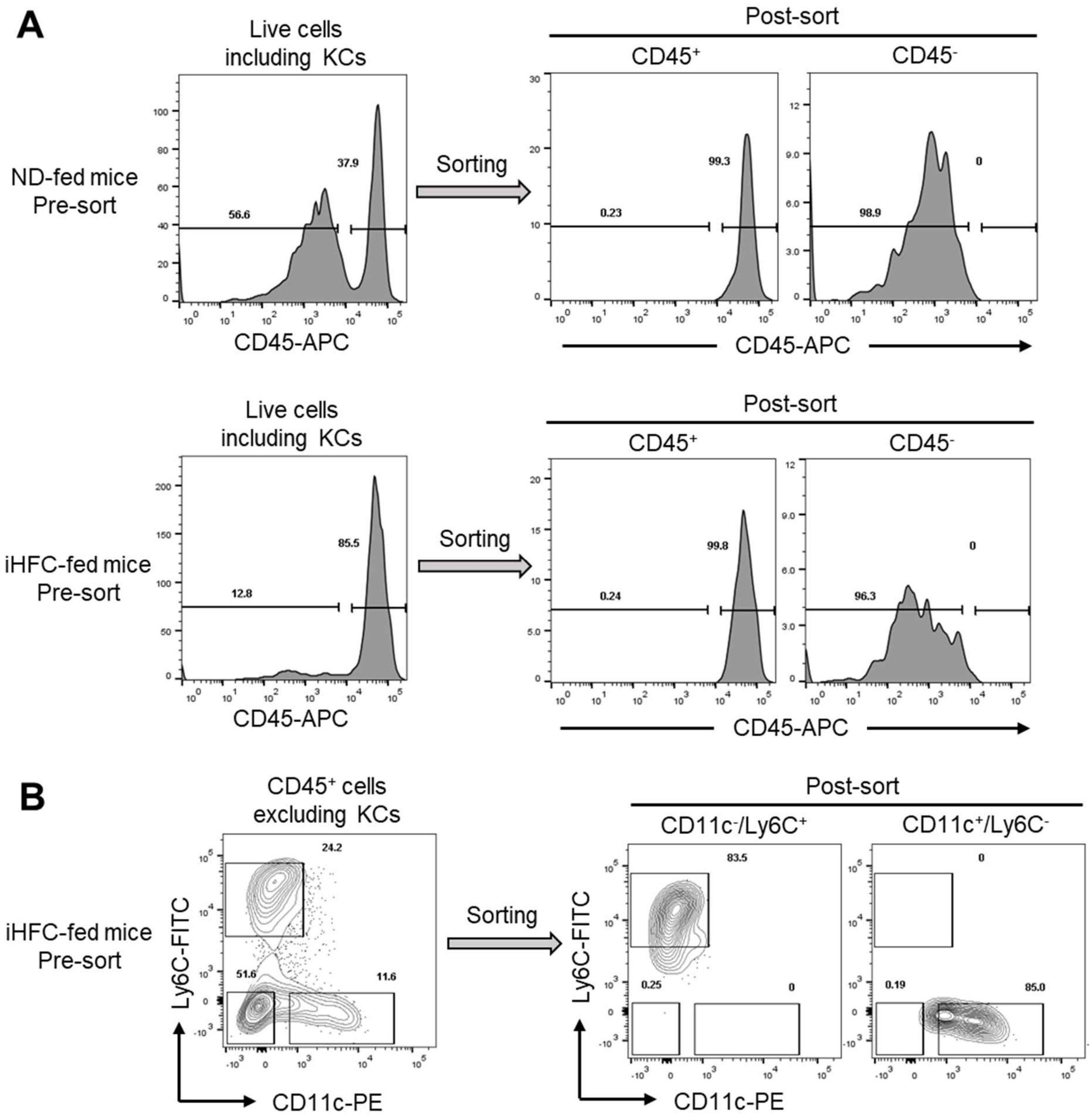

Supplementary Figure S12. Gating strategy for cell sorting of non-parenchymal cell of the liver from TSNO mice.

(A) For the sorting of $CD45^+$ or $CD45^-$ cells from the livers of TSNO mice fed with the ND or iHFC diet for 8 weeks, non-parenchymal cells were stained with APC anti-CD45 antibody and sorted on a FACSMelody. The sort gates and the post-sort analyses are depicted. (B) For the sorting of $CD11c^+/Ly6C^-$ or $CD11c^-/Ly6C^+$ cells from the livers of TSNO mice fed with the iHFC diet for 8 weeks, non-parenchymal cells were stained with APC anti-CD45, FITC anti-Ly6C, and PE anti-CD11c antibodies. $CD45^+$ cells excluding KCs were gated as shown in Figure S4 and then analyzed with a plot of CD11c and Ly6C. $CD11c^+/Ly6C^-$ or $CD11c^-/Ly6C^+$

cells were sorted on a FACSMelody. The sort gates and the post-sort analyses are depicted.
